# Supplementary material for: Bibliometric Review of the Literature on Cone Snail Peptide Toxins from 2000 to 2022
Source: Mar Drugs. 2023 Feb 25;21(3):154. doi: 10.3390/md21030154 (PMC10058278; doi:10.3390/md21030154)
Supplement: Supplementary file 1 [file marinedrugs-21-00154-s001.zip › marinedrugs-2226260-supplementary.pdf]

Supplementary information for

Bibliometric review of the literature on cone snail peptide toxins from 2000 to 2022

Linh T. T. Nguyen[1,2], David J. Craik[1,2], Quentin Kaas[1,2*]

[1] Australian Research Council Centre of Excellence for Innovations in Peptide and Protein Science, The University of Queensland, Brisbane, QLD 4072, Australia
[2] Institute for Molecular Bioscience, The University of Queensland, Brisbane, QLD 4072, Australia
[*] Correspondence: quentin.kaas@gmail.com

Page	Description
2	Code S1: "01-retype.py" Reclassifies the publications as "Article" or "Review" according to PubMed.
4	Code S2: "02-rekeywords.py" Modify keywords to increase consistency. The list of keyword changes are provided in Table S1.
6	Code S3: "03-authors_replace.py" Modify authors first names to increase consistency. The list of first name changes are provided in Table S2.
7	Table S1: List of keyword changes
21	Table S2: List of Authors modifications

Code S1: “01-retype.py” Reclassifies the publications as “Article” or “Review” according to PubMed.

```
import os,sys

# Find pubmed type of each article

pmid = None
pubmed_type = {}
with open('00-data_orig/pubmed-conotoxins.txt') as f:
    for l in f:
        if l.startswith('PMID'):
            if pmid is not None:
                if article is None and review is None:
                    print(f'ERR>could not determine the type of {pmid}')
                pubmed_type[pmid] = 'Review' if review else 'Article'
            pmid = l.strip().split()[1]
            article = review = False
        if l.startswith('PT  - Journal Article'):
            article = True
        if l.startswith('PT  - Review'):
            review = True
pubmed_type[pmid] = 'Review' if review else 'Article'
# Read the list of pubmed used for initial retyping

if os.path.exists('01-retyped/pubmed_types.txt'):
    with open('01-retyped/pubmed_types.txt') as f:
        for l in f:
            F = l.strip().split()
            pubmed_type[F[0]] = F[1]

# Initialise outfile

for filename in ['articles.txt','reviews.txt']:
    filename = f'01-retyped/{filename}'
    with open(filename,'w') as f:
        f.write('FN Clarivate Analytics Web of Science\nVR 1.0\n')
for filename in ['review_changed_to_article.txt','article_changed_to_review.txt']:
    filename = f'01-retyped/{filename}'
    if os.path.exists(filename): os.unlink(filename)
# Reclassifying articles

def sort_wos(wos_def,wos_type,wos_pmid):
    print(wos_pmid)
    if not wos_pmid:
        new_type = wos_type
    elif wos_pmid in pubmed_type:
        new_type = pubmed_type[wos_pmid]

    for i,l in enumerate(wos_def):
        if l.startswith('DT '):
            wos_def[i] = f'DT {new_type}\n'
    wos_def.append('\n')

    filename = 'reviews.txt' if new_type == 'Review' else 'articles.txt'
    with open(f'01-retyped/{filename}','a') as f:
        f.write(''.join(wos_def))
    if wos_pmid:
        if new_type == 'Article' and 'Article' != wos_type:
            with open(f'01-retyped/review_changed_to_article.txt','a') as f:
                f.write(''.join(wos_def))
```

```
        elif new_type == 'Review' and 'Review' != wos_type:
            with open(f'01-retyped/article_changed_to_review.txt','a') as f:
                f.write(''.join(wos_def))
for infile in [f for f in os.listdir('00-data_orig') if f.endswith('.txt') and 'pubmed' not in f ]:
    print(infile)
    wos_def  = None
    wos_type = None
    wos_pmid = None
    has_py = False # if no year then ahead of print
    with open(f'00-data_orig/{infile}') as f:
        for l in f:
            if l.startswith('PT '): # start of article
                if wos_def is not None and has_py:
                    sort_wos(wos_def,wos_type,wos_pmid)
                wos_def = []
                wos_type = wos_pmid = None
                has_py = False
            if l.startswith('PY '):
                has_py = True
            if l.startswith('DT '):
                wos_type = l.strip().split()[1]
                wos_type = 'Review' if wos_type == 'Review' else 'Article'
            if l.startswith('PM '):
                wos_pmid = l.strip().split()[1]
            if l.strip() == '': continue
            if l.startswith('EF'):continue
            if wos_def is not None:
                wos_def.append(l)
    if has_py:
        sort_wos(wos_def,wos_type,wos_pmid)
```

Code S2: "02-rekeywords.py" Modify keywords to increase consistency. The list of keyword changes are provided in Table S1.

```
import sys

def _keys_sorted(dic):
    kws = [kw for kw in dic]
    kws.sort(key=lambda x:dic[x],reverse=True)
    return kws

converts = {}
with open('keywords_changes') as f:
    for l in f:
        kw_from = l[:51].strip()
        kw_to   = l[51:].strip()
        converts[kw_from] = None if kw_to == 'None' else kw_to

def keywords_lines (keywords,header='DE'):
    lines = []
    for w in keywords:
        if not lines: lines = [w]
        elif len(lines[-1])+2+len(w) > 72:
            lines[-1] += ';'
            lines.append(w)
        else: lines[-1] += '; '+w
    return f'{header} '+'\n   '.join(lines)+'\n'
def modify_keywords(filein,fileout):

    kw_de_stats = {}
    kw_id_stats = {}

    in_de = in_id = False
    with open(fileout,'w') as out:
        with open(filein) as f:
            for l in f:
                # DE keywords
                if in_de and l.startswith('  '):
                    de += ' '+l[3:].strip().lower()
                    continue
                if in_de and not l.startswith('  '):
                    de = [i.strip() for i in de.split(';')]
                    de = [kw if kw not in converts else converts[kw] for kw in de \
                        if (kw not in converts or converts[kw] is not None)]
                    de = set(de) # count only a given keyword for each article
                    for kw in de:
                        if kw not in kw_de_stats: kw_de_stats[kw] = 0
                        kw_de_stats[kw] += 1
                    in_de = False
                    out.write(keywords_lines(de,'DE'))
                if l.startswith('DE'):
                    in_de = True
                    de = l[3:].strip().lower()
                    continue
                # ID keywords
                if in_id and l.startswith('  '):
                    idw += ' '+l[3:].strip().lower()
                    continue
                if in_id and not l.startswith('  '):
                    idw = [i.strip() for i in idw.split(';')]
                    idw = [kw if kw not in converts else converts[kw] for kw in idw \
```

```
                if (kw not in converts or converts[kw] is not None)]
            idw = set(idw) # count only a given keyword for each article
            for kw in idw:
                if kw not in kw_id_stats: kw_id_stats[kw] = 0
                kw_id_stats[kw] += 1
            in_id = False
            out.write(keywords_lines(idw,'ID'))
        if l.startswith('ID'):
            in_id = True
            idw = l[3:].strip().lower()
            continue
        out.write(l)
modify_keywords('01-retyped/articles.txt','02-rekeyworded/articles.txt')
modify_keywords('01-retyped/reviews.txt','02-rekeyworded/reviews.txt')
```

Code S3: “03-authors_replace.py” Modify authors first names to increase consistency. The list of first name changes are provided in Table S2.

```
import sys

def split_name(name):
    L = name.split(', ')
    if len(L) == 1: return (name,'')
    L[1].replace('.','')
    if L[0].startswith('De '):
        L[0] = 'de '+L[0][3:]
    if L[1].upper() == L[1]:
        L[1] = L[1].replace(' ','')
    return (L[0].strip(),L[1].strip())

af_replace = {}
with open('af_replace.tab') as f:
    for l in f:
        last_name = l[0:27].strip()
        first_orig = l[27:53].strip()
        first_repl = l[53:].strip()
        if last_name not in af_replace: af_replace[last_name] = {}
        af_replace[last_name][first_orig] = first_repl

def replace_af_list(afs):
    new_af = []
    for af in afs:
        (last,first) = split_name(af)
        if last in af_replace and first in af_replace[last]:
            new_af.append(f'{last}, {af_replace[last][first]}')
        else:
            new_af.append(f'{last}, {first}')
    return new_af

# replace AF

with open('03-author_replace/articles.txt','w') as o:
    with open('02-rekeyworded/articles.txt') as f:
        in_af = False
        article_af = []
        numarticle = 0
        for l in f:
            if l.startswith('AF '):
                in_af  = True
            elif in_af and l[:2] != '  ':
                article_af =replace_af_list(article_af)
                o.write(f'AF {article_af[0]}\n')
                for af in article_af[1:]:
                    o.write(f'   {af}\n')
                article_af = []
                in_af = False
            if in_af:
                article_af.append(l[3:].strip().replace('.',''))
            else:
                o.write(l)

with open('03-author_replace/reviews.txt','w') as o:
    with open('02-rekeyworded/reviews.txt') as f:
        in_af = False
        article_af = []
```

```
        numarticle = 0
        for l in f:
            if l.startswith('AF '):
                in_af  = True
            elif in_af and l[:2] != '  ':
                article_af =replace_af_list(article_af)
                o.write(f'AF {article_af[0]}\n')
                for af in article_af[1:]:
                    o.write(f'   {af}\n')
                article_af = []
                in_af = False
            if in_af:
                article_af.append(l[3:].strip().replace('.',''))
            else:
                o.write(l)

with open('03-author_replace/articles_noAU.txt','w') as o:
    with open('03-author_replace/articles.txt') as f:
        in_au = False
        numarticle = 0
        for l in f:
            if l.startswith('AU '):
                in_au  = True
            elif in_au and l[:2] != '  ':
                in_au = False
            if not in_au:
                o.write(l)
```

Table S1: List of keyword changes

Original Keyword	Replacement Keyword
conotoxin	conotoxins
-conotoxin	conotoxins
conopeptide	conopeptides
cone snail toxin	conopeptides
cone snail toxins	conopeptides
conus peptide	conopeptides
conus peptides	conopeptides
conus-venom peptides	conopeptides
contryphan	contryphans
mu-conotoxin	mu-conotoxins
omega-conotoxin	mu-conotoxins
alpha-conotoxin	alpha-conotoxins
mviia	omega-conotoxin mviia
ziconotide	omega-conotoxin mviia
intrathecal ziconotide	omega-conotoxin mviia
vc1.1	alpha-conotoxin vc1.1
alpha-conotoxin-mii	alpha-conotoxin mii
conotoxin-mii	alpha-conotoxin mii
mii	alpha-conotoxin mii
gi	alpha-conotoxin gi
imi	alpha-conotoxin imi
mi	alpha-conotoxin mi
pnia	alpha-conotoxin pnia
gvia	omega-conotoxin gvia
omega conotoxin gvia	omega-conotoxin gvia
piiia	mu-conotoxin piiia
kiiia	mu-conotoxin kiiia
mu-conotoxin-kiiia	mu-conotoxin kiiia
giiia	mu-conotoxin giiia
mu-conotoxin-giiia	mu-conotoxin giiia
kappa-conotoxin-pviia	kappa-conotoxin pviia
cyclic peptide	cyclic peptides
mouse	None
mice	None
calcium	None
rat	None
peptide	None
peptides	None
nicotine	None
toxin	None
cone snail venom	cone snail venoms
venom	venoms
conus	cone snails
cone snail	cone snails
gastropod genus conus	cone snails
patch-clamp	electrophysiology

patch clamp	electrophysiology
patch-clamp technique	electrophysiology
whole-cell patch clamp	electrophysiology
ion channel	ion channels
nachr	nicotinic acetylcholine receptors
nachrs	nicotinic acetylcholine receptors
nicotinic	nicotinic acetylcholine receptors
nicotinic receptors	nicotinic acetylcholine receptors
nicotinic receptor	nicotinic acetylcholine receptors
nicotinic acetylcholine receptor	nicotinic acetylcholine receptors
acetylcholine	nicotinic acetylcholine receptors
neuronal nicotinic acetylcholine receptor	nicotinic acetylcholine receptors
nicotinic acetylcholine-receptor	nicotinic acetylcholine receptors
nicotinic acetylcholine-receptors	nicotinic acetylcholine receptors
acetylcholine receptor	acetylcholine receptors
acetylcholine-receptor	acetylcholine receptors
acetylcholine-receptors	acetylcholine receptors
calcium channel	voltage-gated calcium channels
calcium channels	voltage-gated calcium channels
ca2+ channels	voltage-gated calcium channels
ca2+ channel	voltage-gated calcium channels
voltage-dependent calcium channels	voltage-gated calcium channels
voltage-dependent ca2+ channels	voltage-gated calcium channels
voltage-gated calcium channel	voltage-gated calcium channels
voltage-dependent calcium channel	voltage-gated calcium channels
voltage gated calcium channel	voltage-gated calcium channels
voltage-sensitive calcium channels	voltage-gated calcium channels
voltage-gated ca2+ channels	voltage-gated calcium channels
voltage-gated ca2+ channel	voltage-gated calcium channels
ca(2+) channel	voltage-gated calcium channels
voltage-activated ca2+ channel	voltage-gated calcium channels
voltage-activated ca2+ channels	voltage-gated calcium channels
vgcc	voltage-gated calcium channels
vgccs	voltage-gated calcium channels
voltage gated calcium channels	voltage-gated calcium channels
t-type	t-type voltage-gated calcium channels
t-type ca2+ channel	t-type voltage-gated calcium channels
t-type ca2+ channels	t-type voltage-gated calcium channels
n-type calcium channels	n-type voltage-gated calcium channels
n-type calcium channel	n-type voltage-gated calcium channels
l-type calcium channel	l-type voltage-gated calcium channels
l-type	l-type voltage-gated calcium channels
l-type ca2+ channel	l-type voltage-gated calcium channels
l-type ca2+ channels	l-type voltage-gated calcium channels
n-type	n-type voltage-gated calcium channels
n-type ca2+ channels	n-type voltage-gated calcium channels
n-type ca2+ channel	n-type voltage-gated calcium channels
p-type	p/q-type voltage-gated calcium channels
p/q-type	p/q-type voltage-gated calcium channels

p/q-type calcium channel	p/q-type voltage-gated calcium channels
p/q-type calcium channels	p/q-type voltage-gated calcium channels
r-type calcium channel	r-type voltage-gated calcium channels
r-type calcium channels	r-type voltage-gated calcium channels
voltage-gated sodium channel	voltage-gated sodium channels
gated sodium-channel	voltage-gated sodium channels
gated sodium-channels	voltage-gated sodium channels
sodium-channels	voltage-gated sodium channels
sodium-channel	voltage-gated sodium channels
sodium channels	voltage-gated sodium channels
sodium channel	voltage-gated sodium channels
voltage gated sodium channel	voltage-gated sodium channels
na+-channels	voltage-gated sodium channels
voltage gated sodium channels	voltage-gated sodium channels
na+ channel	voltage-gated sodium channels
voltage-sensitive sodium channel	voltage-gated sodium channels
voltage-sensitive sodium channels	voltage-gated sodium channels
nmda	n-methyl-d-aspartate receptors
nmda receptor	n-methyl-d-aspartate receptors
nmda receptors	n-methyl-d-aspartate receptors
nmda-receptor	n-methyl-d-aspartate receptors
nmda-receptors	n-methyl-d-aspartate receptors
nmdar	n-methyl-d-aspartate receptors
n-methyl-d-aspartate receptor	n-methyl-d-aspartate receptors
k+ channel	voltage-gated potassium channels
voltage-sensitive potassium channel	voltage-gated potassium channels
voltage-sensitive potassium channels	voltage-gated potassium channels
nmr spectroscopy	nuclear magnetic resonance
nmr	nuclear magnetic resonance
solution structure	nuclear magnetic resonance
nmr structure	nuclear magnetic resonance
nmr structure calculation	nuclear magnetic resonance
nuclear-magnetic-resonance	nuclear magnetic resonance
nmr-spectroscopy	nuclear magnetic resonance
disulfide	disulfide bonds
disulfides	disulfide bonds
disulfide bond	disulfide bonds
disulfide bridge	disulfide bonds
disulfide bridges	disulfide bonds
peptide disulfide	disulfide bonds
peptide disulfides	disulfide bonds
transcriptome	transcriptomes
neuromuscular junction	neuromuscular junctions
neurotoxin	neurotoxins
structure-activity relationship	structure-activity relationships
post-translational modification	post-translational modifications
chromaffin cell	chromaffin cells
agatoxin	agatoxins
support vector machine	support vector machines

calcium current	calcium currents
potassium channel	potassium channels
neurotransmitter	neurotransmitters
conus venom	conus venoms
brain slice	brain slices
sensory neuron	sensory neurons
peptide toxin	peptide toxins
synaptosome	synaptosomes
synapse	synapses
spider toxin	spider toxins
pc12 cell	pc12 cells
analgesic	analgesics
o-superfamily conotoxin	o-superfamily conotoxins
gaba(b) receptor	gaba(b) receptors
calcium channel blocker	calcium channel blockers
ca2+ channel blocker	calcium channel blockers
motor nerve terminal	motor nerve terminals
selenocysteine	selenocysteines
neuropeptide	neuropeptides
catecholamine	catecholamines
antagonist	antagonists
molecular dynamics simulation	molecular dynamics simulations
molecular dynamic	molecular dynamics simulations
molecular dynamics	molecular dynamics simulations
sodium current	sodium currents
ligand-gated ion channel	ligand-gated ion channels
venom peptide	venom peptides
whole-cell recording	whole-cell recordings
dihydropyridine	dihydropyridines
na+/ca2+ exchanger	na+/ca2+ exchangers
acetylcholine-binding protein	acetylcholine-binding proteins
d-amino acid	d-amino acids
hippocampal neuron	hippocampal neurons
ca2+ current	ca2+ currents
sympathetic nerve	sympathetic nerves
conomarphin	conomarphins
receptor	receptors
conantokin	conantokins
conorfamide	conorfamides
opioid	opioids
peptidomimetic	peptidomimetics
neuronal nicotinic receptor	neuronal nicotinic receptors
signal peptide	signal peptides
excitatory junction potential	excitatory junction potentials
conotoxin precursor	conotoxin precursors
alpha 6 subunit	alpha 6 subunits
g-protein	g-proteins
mollusc	molluscs
cyclotide	cyclotides

antiepileptic drug	antiepileptic drugs
channel blocker	channel blockers
active zone	active zones
synaptic vesicle	synaptic vesicles
ryanodine receptor	ryanodine receptors
ng108-15 cell	ng108-15 cells
inhibitor	inhibitors
xenopus oocyte	xenopus oocytes
oocyte	oocytes
peptide conformation	peptide conformations
glycopeptide	glycopeptides
marine toxin	marine toxins
mglur	mglurs
antimicrobial peptide	antimicrobial peptides
alpha 3 beta 4 nachr	alpha 3 beta 4 nachrs
thiol	thiols
afferent	afferents
bk channel	bk channels
cholinergic interneuron	cholinergic interneurons
pain model	pain models
n-type calcium channel blocker	n-type calcium channel blockers
p2y receptor	p2y receptors
orexin	orexins
pyramidal neuron	pyramidal neurons
tachykinin	tachykinins
scorpion toxin	scorpion toxins
disulfide-rich peptide	disulfide-rich peptides
epsc	epscs
nerve terminal	nerve terminals
seizure	seizures
alpha 9 alpha 10 nachr	alpha 9 alpha 10 nachrs
neuron	neurons
monkey	monkeys
nicotinic receptor subtype	nicotinic acetylcholine receptor subtypes
nicotinic receptor subtypes	nicotinic acetylcholine receptor subtypes
m-superfamily conotoxin	m-superfamily conotoxins
histamine h-3 receptor	histamine h-3 receptors
neuronal network	neuronal networks
potassium current	potassium currents
gpcr	gpcrs
calcium channel antagonist	calcium channel antagonists
dopaminergic neuron	dopaminergic neurons
t-superfamily conotoxin	t-superfamily conotoxins
cerebrocortical synaptosome	cerebrocortical synaptosomes
nociceptor	nociceptors
antioxidant	antioxidants
dorsal root ganglion neuron	dorsal root ganglion neurons
synthetic peptide	synthetic peptides
mu-opioid receptor	mu-opioid receptors

neurotrophic factor	neurotrophic factors
motoneuron	motoneurons
n-type voltage-gated calcium channel	n-type voltage-gated calcium channels
secondary structure	secondary structures
alpha-neurotoxin	alpha-neurotoxins
girk channel	girk channels
membrane protein type	membrane protein types
cannabinoid	cannabinoids
oscillation	oscillations
hydrophobicity pattern	hydrophobicity patterns
ampa receptor	ampa receptors
species interaction	species interactions
cerebellar granule cell	cerebellar granule cells
gaba(a) receptor	gaba(a) receptors
protein phosphatase	protein phosphatases
repetitive action potential	repetitive action potentials
transcriptomic	transcriptomics
proteomic	proteomics
bioinformatic	bioinformatics
trypsin inhibitor	trypsin inhibitors
protein kinase	protein kinases
anticonvulsant	anticonvulsants
phorbol ester	phorbol esters
nematocyst	nematocysts
omega-toxin	omega-toxins
cerebellar granule neurone	cerebellar granule neurones
xenopus laevis oocyte	xenopus laevis oocytes
cb1 receptor	cb1 receptors
calcium channel subtype	voltage-gated calcium channel subtypes
calcium channel subtypes	voltage-gated calcium channel subtypes
excitotoxin	excitotoxins
molecular model	molecular models
assassin bug	assassin bugs
enkephalin	enkephalins
sprague-dawley rat	sprague-dawley rats
conoprotein	conoproteins
homology model	homology models
calcium oscillation	calcium oscillations
point mutation	point mutations
nmda antagonist	nmda antagonists
nicotinic agonist	nicotinic agonists
calcium antagonist	calcium antagonists
alpha 6 nachr	alpha 6 nachrs
beta subunit	beta subunits
hippocampal synaptosome	hippocampal synaptosomes
calcium channels blocker	calcium channels blockers
excitatory amino acid	excitatory amino acids
dopamine receptor	dopamine receptors
mimetic	mimetics

primary neuronal culture	primary neuronal cultures
hyaluronidase	hyaluronidases
neuronal culture	neuronal cultures
alpha 7 subunit	alpha 7 subunits
cholinergic neuron	cholinergic neurons
voltage-gated ion channel	voltage-gated ion channels
subtype	subtypes
astrocyte	astrocytes
spinal neuron	spinal neurons
excitatory peptide	excitatory peptides
pyrethroid	pyrethroids
cephalopod	cephalopods
pyrazole	pyrazoles
enzyme	enzymes
a(1) adenosine receptor	a(1) adenosine receptors
protecting group	protecting groups
subfornical organ	subfornical organs
corticostriatal afferent	corticostriatal afferents
n-type voltage-dependent calcium channel	n-type voltage-dependent calcium channels
voltage-gated channel	voltage-gated channels
ionic channel	ionic channels
linear peptide	linear peptides
waglerin	waglerins
muscle relaxant	muscle relaxants
binding site	binding sites
binding assay	binding assays
ion channel blocker	ion channel blockers
sk channel	sk channels
ba2+ current	ba2+ currents
alpha(2)-adrenoceptor	alpha(2)-adrenoceptors
muscarinic receptor	muscarinic receptors
a(1) receptor	a(1) receptors
trigeminal neuron	trigeminal neurons
metabotropic glutamate receptor	metabotropic glutamate receptors
melanotrope	melanotropes
nmda receptor subunit	nmda receptor subunits
voltage-dependent calcium channel	voltage-dependent calcium channel
antagonist	antagonists
juvenile hormone acid	juvenile hormone acids
adrenal chromaffin cell	adrenal chromaffin cells
ca2+-activated k+ channel	ca2+-activated k+ channels
presynaptic ca2+ channel	presynaptic ca2+ channels
high-voltage-activated ca2+ channel	high-voltage-activated ca2+ channels
l-type voltage-dependent ca2+ channel	l-type voltage-dependent ca2+ channels
p2x receptor	p2x receptors
ionic liquid	ionic liquids
muscle-type nachr	muscle-type nachrs
kcnq channel	kcnq channels
knottin	knottins

polynucleotide	polynucleotides
g protein	g proteins
alpha d-conotoxin	alpha d-conotoxins
potassium channel blocker	potassium channel blockers
agonist	agonists
calcium ion channel	calcium ion channels
fluorescent probe	fluorescent probes
alpha 3 beta 2 nachr	alpha 3 beta 2 nachrs
alpha 4 beta 2 nachr	alpha 4 beta 2 nachrs
acetylcholinesterase	acetylcholinesterases
marine cone snail	marine cone snails
primary culture	primary cultures
ca2+ uptake	ca2+ uptakes
crystal-structure	crystal-structures
protein	proteins
brain	brains
subunit	subunits
mechanism	mechanisms
channel	channels
ion-channel	ion-channels
sequence	sequences
skeletal-muscle	skeletal-muscles
calcium-channel	calcium-channels
model	models
3-dimensional structure	3-dimensional structures
blocker	blockers
binding-site	binding-sites
block	blocks
site	sites
secretion	secretions
calcium-channel blocker	calcium-channel blockers
messenger-rna	messenger-rnas
guinea-pig	guinea-pigs
snail	snails
system	systems
subunit messenger-rna	subunit messenger-rnas
ligand	ligands
beta-subunit	beta-subunits
force-field	force-fields
database	databases
program	programs
binding protein	binding proteins
disease	diseases
gene	genes
d-aspartate receptor	d-aspartate receptors
neuromuscular-junction	neuromuscular-junctions
membrane	membranes
funnel-web spider	funnel-web spiders
precursor	precursors

calcium-channel antagonist	calcium-channel antagonists
domain	domains
drug	drugs
delta-conotoxin	delta-conotoxins
intraspecific variation	intraspecific variations
posttranslational modification	posttranslational modifications
cholinergic-receptor	cholinergic-receptors
contraction	contractions
capsaicin receptor	capsaicin receptors
duct	ducts
amino-acid-sequence	amino-acid-sequences
growth-factor	growth-factors
target	targets
pathway	pathways
residue	residues
plasma-membrane	plasma-membranes
chemical-shift	chemical-shifts
cell	cells
bond	bonds
sensitive calcium-channel	sensitive calcium-channels
shaker k+ channel	shaker k+ channels
receptor antagonist	receptor antagonists
binding-protein	binding-proteins
algorithm	algorithms
tool	tools
sea-anemone	sea-anemones
cation channel	cation channels
animal-model	animal-models
cell-line	cell-lines
simulation	simulations
envenomation	envenomations
motif	motifs
amino-acid	amino-acids
ion	ions
fusion protein	fusion proteins
structural motif	structural motifs
marine gastropod	marine gastropods
auxiliary subunit	auxiliary subunits
k+ current	k+ currents
mutant	mutants
after-hyperpolarization	after-hyperpolarizations
receptor agonist	receptor agonists
aqueous-solution	aqueous-solutions
neurokinin-1 receptor	neurokinin-1 receptors
pattern	patterns
scaffold	scaffolds
coiled-coil	coiled-coils
superfamily conotoxin	superfamily conotoxins
increase	increases

activated protein-kinase	activated protein-kinases
sea-anemone toxin	sea-anemone toxins
therapeutic target	therapeutic targets
alpha-7 subunit	alpha-7 subunits
vanilloid receptor	vanilloid receptors
spider venom	spider venoms
molecular-dynamics simulation	molecular-dynamics simulations
region	regions
salivary-gland	salivary-glands
inward current	inward currents
alpha(1a) subunit	alpha(1a) subunits
origin	origins
na+-channel	na+-channels
k+-channel	k+-channels
peptide ligand	peptide ligands
population	populations
dependent ca2+ channel	dependent ca2+ channels
polypeptide	polypeptides
competitive antagonist	competitive antagonists
nmda receptor antagonist	nmda receptor antagonists
vaccine	vaccines
snake-venom	snake-venoms
charge	charges
gated potassium channel	gated potassium channels
nerve	nerves
state	states
modulator	modulators
cholinergic receptor	cholinergic receptors
protein-coupled receptor	protein-coupled receptors
end-plate	end-plates
dependent k+ channel	dependent k+ channels
protease inhibitor	protease inhibitors
form	forms
alpha-1 subunit	alpha-1 subunits
membrane-protein	membrane-proteins
voltage sensor	voltage sensors
dendrotoxin	dendrotoxins
h-3 receptor	h-3 receptors
circular protein	circular proteins
alpha(1) subunit	alpha(1) subunits
sympathetic neuron	sympathetic neurons
beta-3 subunit	beta-3 subunits
line	lines
neuronal calcium-channel	neuronal calcium-channels
neonatal-rat	neonatal-rats
isoform	isoforms
pain pathway	pain pathways
lanthionine	lanthionines
discharge	discharges

na+ current	na+ currents
movement	movements
muscle sodium-channel	muscle sodium-channels
transition	transitions
neurodegenerative disease	neurodegenerative diseases
alpha-subunit	alpha-subunits
contribute	contributes
chain	chains
induced relaxation	induced relaxations
glioma	gliomas
positive allosteric modulator	positive allosteric modulators
reveal	reveals
macrophage	macrophages
calcium-binding protein	calcium-binding proteins
mutation	mutations
amino-acid substitution	amino-acid substitutions
subunit gene	subunit genes
substitution	substitutions
potassium conductance	potassium conductances
protect	protects
alpha-6 subunit	alpha-6 subunits
alpha-like toxin	alpha-like toxins
tissue	tissues
cannabinoid receptor	cannabinoid receptors
presynaptic calcium current	presynaptic calcium currents
ca-2+ channel	ca-2+ channels
dynamics simulation	dynamics simulations
subtype-selective ligand	subtype-selective ligands
trp channel	trp channels
excitatory amino-acid	excitatory amino-acids
store	stores
route	routes
insight	insights
release channel	release channels
integrin	integrins
ab-initio calculation	ab-initio calculations
hydrophobic interaction	hydrophobic interactions
designed peptide	designed peptides
potential wave-form	potential wave-forms
gated na+ channel	gated na+ channels
p2y(1) receptor	p2y(1) receptors
protease	proteases
binding domain	binding domains
clinical-trial	clinical-trials
projection	projections
gastropod	gastropods
bridge	bridges
hydrogen-bond	hydrogen-bonds
injection	injections

heat	heats
activated potassium channel	activated potassium channels
proton	protons
mossy fiber	mossy fibers
ca2+-activated k+ current	ca2+-activated k+ currents
interface	interfaces
chloride channel	chloride channels
adrenoceptor	adrenoceptors
polymorphism	polymorphisms
ribbon synapse	ribbon synapses
cardiovascular-disease	cardiovascular-diseases
fluctuation	fluctuations
acetylcholine-receptor antagonist	acetylcholine-receptor antagonists
transient	transients
gtp-binding protein	gtp-binding proteins
precursor protein	precursor proteins
allosteric interaction	allosteric interactions
culture	cultures
define	defines
component	components
probe	probes
extract	extracts
k-atp channel	k-atp channels
barrier hydrogen-bond	barrier hydrogen-bonds
beta-hairpin	beta-hairpins
activated calcium-channel	activated calcium-channels
frog embryo	frog embryos
chain fatty-acid	chain fatty-acids
protein-protein interaction	protein-protein interactions
neural-network	neural-networks
linkage	linkages
decrease	decreases
5-ht3 receptor	5-ht3 receptors
alpha-adrenoceptor	alpha-adrenoceptors
module	modules
variant	variants
trpv1 receptor	trpv1 receptors
support	supports
c-fiber	c-fibers
analog	analogs
protein conformation	protein conformations
bungarotoxin binding-site	bungarotoxin binding-sites
polyamine	polyamines
pancreatic beta-cell	pancreatic beta-cells
conformational-change	conformational-changes
t-superfamily conotoxins	t-superfamily
conotoxin superfamily	superfamily
superfamily conotoxin	superfamily
superfamily conotoxins	superfamily

expression	None
identification	None
diversity	None
modulation	None
mechanisms	None
systems	None
na+	None
sodium	None
calcium	None
water	None

Table S2: List of Authors modifications

Last Name	Original Last Name	Replacement Last Name
Abdel-Rahman	Mohamed	Mohamed A
Abrahamsen	B	Bjarke
Acuna-Castroviejo	D	Dario
Adachi-Akahane	S	Satomi
Adams	DA	Denise A
Adams	DJ	David J
Adams	David	David J
Adams	David J	David J
Adams	David John	David J
Adams	Denise	Denise A
Adamson	KJ	Kevin J
Addy	Nii	Nii A
Aguanno	S	Salvatore
Aguilar	J	Justo
Aguilar	MB	Manuel B
Aguilar	Manuel	Manuel B
Agwa	Akello	Akello J
Ahn	Duck -Sun	Duck-Sun
Ait-Ghezala	G	Ghania
Akaike	N	Norio
Akbarali	Hamid	Hamid I
Akerman	S	Simon
Akita	T	Tenpei
Akiyama	T	Tsuyoshi
Akiyoshi	T	Takeshi
Akondi	Kalyana	Kalyana Bharati
Akondi	Kalyana B	Kalyana Bharati
Akondi	KB	Kalyana Bharati
Al-Sabi	A	Ahmed
Al-Sabi	AHMED	Ahmed
Albillos	A	Almudena
Alburae	Najla	Najla Ali
Aldea	M	Marcos
Alewood	D	Dianne
Alewood	P	Paul F
Alewood	PF	Paul F
Alewood	P F	Paul F
Alewood	Paul	Paul F
Alewood	Paul Francis	Paul F
Alfaro	Michael	Michael E
Alfonso	M	Miguel
Alger	B	Bradley E
Allen	CN	Charles N
Allen	Jeff	Jeffrey W
Almanza	A	Angelica
Almaraz	L	Laura

Althaus	M	Mike
Alvarez	J	Javier
Andersen	Tim	Timothy L
Andersen	Timothy	Timothy L
Anderson	DJ	David J
Anderson	Matthew	Matthew P
Anderson	Tatiana	Tatiana M
Andreasen	Jesper	Jesper Tobias
Andres-Mateos	E	Eva
Andresen	Michael	Michael C
Andrews	Robert K	Robert Keith
Angus	JA	James A
Angus	James	James A
Annunziato	L	Lucio
Apte	Deepak	Deepak Arun
Arbour	Christine	Christine A
Ardiles	Alvaro	Alvaro O
Arellano	Rogelio O.	Rogelio O
Argiolas	A	Antonio
Ariano	P	Paolo
Armishaw	C	Christopher J
Armishaw	Chris J	Christopher J
Armishaw	Christopher	Christopher J
Aronheim	A	Ami
Arreola	JL	Jose Luis
Artalejo	Antonio R	Antonio R
Ascenzi	P	Paolo
Astashev	Maxim E	Maksim E
Atchison	WD	William D
Atkins	A	Annette
Atkinson	RA	Robert Andrew
Audsley	N	Neil
Augustijns	Patrick	Patrick F
Ayabe	T	Tokiyoshi
Ayers	JT	Joshua T
Azam	L	Layla
Bacci	A	Alberto
Baell	JB	Jonathan B
Baell	Jonathan	Jonathan B
Bagalkot	Tarique	Tarique R
Baker	Margaret	Margaret R
Balaram	P	Padmanabhan
Balashevich	TV	Tatjana
Baldelli	P	Pietro
Ballard	C	Clive
Balsara	Rashna	Rashna D
Band-Schmidt	Christine J	Christine Johanna
Bandyopadhyay	P	Pradip K
Bandyopadhyay	PK	Pradip K

Bandyopadhyay	Pradip	Pradip K
Bansal	P	Paramjit S
Bansal	PS	Paramjit S
Barahona	MV	Maria Victoria
Barahona	Ma Victoria	Maria Victoria
Barbier	J	Julien
Barg	S	Sebastian
Bargas	J	Jose
Barnes	S	Steven
Barral	J	Jaime
Barran	Perdita	Perdita E
Basheer	Soorej	Soorej M
Baskin	Robert P	Robert Paul
Bathgate	Ross	Ross A D
Bathgate	RA	Ross A D
Batista	Cesar	Cesar V F
Baucum	Anthony	Anthony J
Baufreton	J	Jerome
Bautista	W	Wendy
Bayrhuber	M	Monika
Bean	BP	Bruce P
Beani	L	Lorenzo
Beattie	R	RE
Beaudet	A	AL
Becerril	B	Baltazar
Becker	A	Albert J
Becker	S	Stefan
Becq	F	Frederic
Beedle	AM	Aaron M
Beedle	Aaron	Aaron M
Beirao	PSL	Paulo S L
Beirao	Paulo	Paulo S L
Bencherif	M	Merouane
Benedito	S	Sara
Benham	CD	Christopher D
Benoit	E	Evelyne
Berecki	G	Geza
Berezhnov	Alexey	Alexey V
Berman	RF	Robert F
Berretta	N	Nicola
Bertrand	D	Daniel
Bertrand	Sandrine	Sandrine S
Bharadwaj	Vivek	Vivek S
Bhatia	S	Swapnil
Bian	X	Xiaochun
Bian	XC	Xiaochun
Bianciotti	LG	Liliana Graciela
Biass	D	Daniel
Biggs	Jason	Jason S

Biller	Anna	Anna Magdalena
Binda	Nancy	Nancy Scardua
Binda	Nancy S	Nancy Scardua
Bingham	J-P	Jon-Paul
Bingham	JP	Jon-Paul
Birembaut	P	Philippe
Bischofberger	J	Josef
Bisschops	R	Raf
Biswal	Himansu S	Himansu S
Bito	H	Haruhiko
Bjorkqvist	M	Maria
Blanchfield	J	Joanne T
Blanchfield	JT	Joanne T
Blanck	TJJ	Thomas J J
Blanck	Thomas	Thomas J J
Blandizzi	C	Corrado
Blom	AM	Anna M
Blumcke	I	Ingmar
Boccaccio	A	Anna
Boeckxstaens	Guy	Guy E
Boehm	S	Stefan
Boelens	R	Rolf
Bohr	I	Iwo
Bohr	IJ	Iwo
Boisbouvier	J	Jerome
Bolam	JP	J Paul
Bonsi	P	Paola
Bony	Anuja	Anuja R
Bordia	T	Tanuja
Borges	Marcia H	Marcia Helena
Borges	MH	Marcia Helena
Bornstein	Joel	Joel C
Borrelli	F	Francesca
Boschen	Karen	Karen E
Bose	U	Utpal
Bosmans	F	Frank
Bosse	Gabriel	Gabriel D
Bourne	Y	Yves
Bouryi	Vitali	Vitali A
Brailoiu	E	Eugen
Brain	KL	Keith L
Brain	Keith	Keith L
Branca	Jacopo	Jacopo Juno Valerio
Breedlove	S. Marc	S Marc
Brehm	P	Paul
Brierley	Stuart	Stuart M
Brimble	Margaret	Margaret A
Brock	JA	James A
Brock	James	James A

Brookes	S	SJH
Brookes	SJ	SJH
Brown	Robert	Robert W B
Broxton	N	NM
Brunzell	DH	Darlene H
Bubis	J	Jose
Buczek	O	Olga
Buczek	P	Pawel
Buehler	Markus J.	Markus J
Bugianesi	RM	Randal M
Buhlman	LM	Lori M
Buhlman	Lori	Lori M
Bulaj	G	Grzegorz W
Bulaj	Grzegorz	Grzegorz W
Bulaj	Grzeorz	Grzegorz W
Bunn	SJ	Stephen J
Bunnett	NW	Nigel W
Buratini	J	Jose
Buschard	K	Karsten
Butler	Margi	Margi I
Cabalteja	Chino	Chino C
Cabang	April	April B
Cabot	PJ	Peter John
Cabot	Peter J	Peter John
Caceres	AI	Ana I
Calabresi	P	Paolo
Calixto	JB	Joao Batista
Callaghan	B	Brid P
Callaghan	Brid	Brid P
Campbell	Adrian	Adrian P
Campos	Maria	Maria Martha M
Cannon	Jason	Jason R
Cano-Abad	Maria F.	Maria F
Cano-Abad	MF	Maria F
Cano-Abad	Maria	Maria F
Canzoniero	LMT	Lorella Maria Teresa
Cao	J	Jinshan
Cao	Zhijian	Zhi-Jian
Capasso	R	Raffaele
Capogna	M	Marco
Carbone	E	Emilio
Cardenas	AM	Ana Maria
Cardoso	Valbert N	Valbert Nascimento
Cardoso	FC	Fernanda C
Carlomagno	T	Teresa
Carrera	P	Paola
Carrillo-Reid	L	Luis
Carroll	FI	F Ivy
Carta	M	Mario

Carvalho	AP	Arselio P
Carvalho	CM	Caetana M
Castellino	FJ	Francis J
Castiglioni	AJ	Andrew J
Castillo	C	Cecilia
de Castro	Celio J	Celio Jose
de Castro	Cello J	Celio Jose
de Castro	CJ	Celio Jose
Cater	Heather	Heather L
Catlin	P	Philip
Celie	PH	PHN
Cellek	S	Selim
Cembella	Allan	Allan D
Centonze	D	Diego
Cervantes-Luevano	Karla	Karla E
Cervantes-Luevano	KE	Karla E
Cervetto	C	Chiara
Chagot	B	Benjamin
Chaim	Olga	Olga Meiri
Chalon	S	Sylvie
Chan	Lai Y	Lai Yue
Chang	Yongchang	Yong-chang
Changeux	J-P	Jean-Pierre
Changeux	JP	Jean-Pierre
Chapman	V	Victoria
Charnet	P	Pierre
Cha	SK	Seung-Kyu
Chen	Dejie	De-jie
Chen	Hanchun	Han-chun
Chen	Jisheng	Ji-Sheng
Chen	Zongyun	Zong-Yun
Cheng	Q	Qiong
Chernyavsky	Alexander	Alexander I
Chevaleyre	V	Vivien
Chew	Lindsey	Lindsey A
Chi	Chengwu	Cheng-wu
Chi	Cheng-Wu	Cheng-wu
Chin	Yanni	Yanni K -Y
Choudhary	G	Gaurav
Chougule	N	Nanasaheb
Christensen	Sean	Sean B
Christoph	P	Palle
Christophersen	P	Palle
Chudzinski-Tavassi	Ana M	Ana M.
Chuhma	N	Nao
Chun	Joycelyn B	Joycelyn B S
Chung	JM	Jong Min
Cho	J-H	Jin-Hwa
Cho	JH	Jin-Hwa

Choi	I-S	In-Sun
Christie	MJ	MacDonald J
Christie	Macdonald J	MacDonald J
Chugunov	AO	Anton O
Cohen	BN	Bruce N
Ciccarelli	A	Alessandro
Cilleros	Vctor	Victor
Cizkova	D	Dasa
Clark	RJ	Richard J
Clark	Richard	Richard J
Clementi	F	Francesco
Cobley	Claire M.	Claire M
Colgrave	ML	Michelle L
Colgrave	Michelle	Michelle L
Colless	B	Barbara
Colli-Dula	Reyna C	Reyna Cristina
Collier	Abby	Abby C
Collins	AC	Allan C
Collins	Mark	Mark O
Compain	P	Philippe
Conceicao	IM	Isaltino Marcelo
Conceicao	Isaltino M	Isaltino Marcelo
Conibear	Anne	Anne C
Connolly	J	Jacklyn
Conyers	C	Chris
Cooke	I	Ian M
Cooke	Ian	Ian M
Cooper	J	John F
Cooper	Matthew A.	Matthew A
Copeland	RL	Robert L
Copeland	Robert	Robert L
Cordeiro	MN	Marta Nascimento
Cordeiro	Marta N	Marta Nascimento
Cordeiro	Marta do N	Marta Nascimento
Cordeiro	Marta do Nascimento	Marta Nascimento
Cordeiro	Sonke	Soenke
Cordova	Marco	Marco A
Cornil	Charlotte A	Charlotte Anne
Correia-De-Sa	P.	Paulo
Correia-De-Sa	P	Paulo
Correia-de-Sa	P	Paulo
Cortez	LM	Leonardo
Cory	JS	Jenny S
Corzo	G	Gerardo
Costa	C	Cinzia
Costa	G	Gonzalo
Costa	M	Marcello
Costa	Pedro M	Pedro M.
Cragg	Stephanie	Stephanie J

Cragg	SJ	Stephanie J
Craig	PJ	Patricio O
Craig	P	Patricio O
Craik	D	David J
Craik	D J	David J
Craik	DJ	David J
Craik	David	David J
Croker	DE	Daniel E
Croker	Daniel	Daniel E
Crooks	PA	Peter A
Cruz	LJ	Lourdes J
Cruz	Lourdes	Lourdes J
Cruz	Ronald Allan	Ronald Allan L
Cui	Jibin	Ji-Bin
Cumming	P	Paul
Cummins	Scott	Scott F
Cummins	SF	Scott F
Cummins	TR	Theodore R
Cunha	Thiago M	Thiago Mattar
Currie	Kevin P	Kevin P M
Curro	D	Diego
D'Abramo	M	Marco
Dai	Peng	Pingli
Dai	Qiuyun	Qiu-Yun
Dai	QY	Qiu-Yun
Dai	X	Xiandong
Dai	XD	Xiandong
Dale	Camila	Camila S
Dale	N	Nicholas
Dalmolin	Gerusa D	Gerusa Duarte
Dalmolin	Geruza	Gerusa Duarte
Daly	NL	Norelle L
Daly	Norelle	Norelle L
Damaj	MI	M Imad
Damjanovich	S	Sandor
Dani	JA	John A
Dave	JR	Jitendra R
David	Adam	Adam Z
Davies	Alexander	Alexander J
Dawson	LA	Lee A
Day	M	Margot
Dayanithi	G	Govindan
Deaciuc	AG	Agripina G
Deak	F	Ferenc
Degenaar	P	Patrick
Demel	SL	Stacie L
Del Tacca	M	MD
Delaney	KR	Kerry R
Delgado-Lezama	R	Rodolfo

Denisov	Stepan	Stepan S
Dergacheva	O	Olga
Deuis	Jennifer	Jennifer R
Di Angelantonio	S	Silvia
Di Marzo	V	Vincenzo
Di Monte	Donato	Donato A
Diamond	Jeffrey	Jeffrey S
Diaz	Mary	Mary E
Dickenson	AH	Anthony H
Dickson	Suzanne	Suzanne L
Diener	M	Martin
Dierssen	M	Mara
Ding	Jiuping	Jiu-Ping
Dirnagl	U	Ulrich
Dissing	S	Steen
Distasi	C	Carla
Dobrev	D	Dobromir
Dodd	PR	Peter Ronald
Doering	C	CJ
Dolezal	V	Vladimir
Dolgikh	Dmitry	Dmitry A
Dolphin	AC	Annette C
Donaldson	Lucy	Lucy F
Donevan	S	SD
Dong	F	Fangting
Dong	H	Hua-Jin
Donly	C	Cam
Dos Santos	RG	Raquel Gouvea
Douville	Renee	Renee N
Dowell	C	Cheryl D
Dowell	CD	Cheryl D
Dowell	Cheryl	Cheryl D
Down	J	JG
Drenan	RM	Ryan M
Drinkwater	R	Roger
Drucker	DJ	Daniel J
Du	Weihong	Wei-Hong
Duarte	CB	Carlos B
Duda	TF	Thomas F
Dudley	SC	Samuel C
Dudley	Samuel	Samuel C
Dudley	Samuel C	Samuel C
Duggan	PJ	Peter J
Dun	NJ	Nae J
Duran	R	Rafael
Duran-Riveroll	Lorena	Lorena M
Durnin	L	Leonie
Dutertre	S	Sebastien
Dutton	J	Julie L

Dutton	JL	Julie L
Dutton	Julie	Julie L
Dwoskin	LP	Linda P
Dy	CY	Catherine Y
Dy	Catherine	Catherine Y
Edwards	Jeff	Jeffrey G
Efremov	RG	Roman G
Egorova	Natalia S	Natalya S
Ekberg	J	Jenny
Ekberg	JA	Jenny
Eksteen	J Johannes	Jacobus Johannes
El Ayadi	A	Amina
El-Seedi	Hesham R.	Hesham R
Elgorban	Abdallah	Abdallah M
Eliasson	L	Lena
Eliseo	T	Tommaso
Elliger	C	C A
Elliger	CA	C A
Ellinor	Patrick	Patrick T
Ellison	M	Michael
Elmslie	KS	Keith S
Elmslie	Keith	Keith S
Emidio	Nayara B	Nayara Braga
Engel	JA	Jorgen A
Engel	Jorgen	Jorgen A
Engisch	KL	Kathrin L
Engle	Staci	Staci E
Engle	SE	Staci E
Ennes	HS	Helena S
Escames	G	Germaine
Escoubas	P	Pierre
Espino	Samuel	Samuel S
Esquerda	Josep	Josep E
Estephan	R	Rima
Estrada	Juan	Juan A
Evans	Edward	Edward R J
Exley	R	Richard
Fainzilber	M	Mike
Falcon	A	Andres
Fan	Chongxu	Chong-Xu
Fan	CX	Chong-Xu
Fan	H	Hong
Fan	Yingxu	Yong-Xian
Fang	Gemin	Ge-Min
Fang	Y	Yaping
Faria	M	Miguel
Farrell	Spring	Spring R
Farrugia	G	Gianrico
Favreau	P	Philippe

Favreau	Ph	Philippe
Favreau	Philipe	Philippe
Fawley	Jessica	Jessica A
Fedosov	Alexander	Alexander E
Felicori	Liza F	Liza Figueiredo
Felix	R	Ricardo
Feng	Jianchen	Jiancheng
Feng	Yonge	Yuchao
Ferber	M	Michael
Ferguson	AV	Alastair V
Fernandes	Vitor S	Vitor Samuel
Ferreira	J	Juliano
Ferreira	Renata C M	Renata Cristina Mendes
Ferris	Mark	Mark J
Ferris	MJ	Mark J
Fiedler	B	Brian
Fields	GB	Gregg B
Filchakova	Olena	Olena M
Finley	Michael	Michael F A
Finol-Urdaneta	Rocio K.	Rocio K
Fischer	H	Harald
Fleck	Roland	Roland A
Flinspach	M	Mack
Floran	B	Benjamin
Flores-Soto	E	Edgar
Foehring	Robert	Robert C
Forni	PE	Paolo E
Forni	Paolo E.	Paolo E
Forsyth	SA	Stewart A
Forti	L	Lia
Fouda	Maged	Maged M A
Fowler	Christie	Christie D
Fozzard	HA	Harry A
Fragale	A	Alessandra
Franchini	A	Antonella
Franklin	Jayaseelan B	Jayaseelan Benjamin
Fredriksson	R	Robert
Freiman	T	Thomas
Freiman	TM	Thomas
French	RJ	Robert J
Fricker	D	Desdemona
Fronius	M	Martin
Fruttero	R	Roberta
Fujita	S	Shuji
Fukushima	Fabiola B	Fabiola Bono
Furie	BC	Barbara C
Furness	JB	John B
Furukawa	K	Ken-Ichi
Fuxe	K	Kjell

Gable	RW	Robert W
Gable	Robert	Robert W
Gagliardi	RJ	Rubens J
Galanis	Athanassios	Athanassios S
Galanis	Athanassios S	Athanassios S
Galanis	Athanassios S.	Athanassios S
Galarraga	E	Elvira
Gale	Jonathan	Jonathan E
Galea	Charles	Charles A
Gallagher	OP	Oliver P
Galligan	JJ	James J
Galvan	E	Emilio J
Galvan	Emilio	Emilio J
Gandia	L	Luis
Gao	Bingmiao	Bing-Miao
Gao	F	Fen-fei
Gao	Fenfei	Fen-fei
Garcia	AG	Antonio G
Garcia	Alfredo J	Alfredo J
Garcia	David	David E
Garcia	E	Edgar
Garcia	J	Javier
Garcia	N	Neus
Garcia-Pascual	A	Angeles
Garcia-Rill	E	Edgar
Garcia-Sacristan	A	Albino
Garcia-Sancho	J	Javier
Gardner	A	Alice
Garrett	JE	James E
Gasparini	S	Sonia
Gautier	Helene	Helene O B
Gaydukov	A	Alexander
Gayler	K	KR
Geiger	James	James H
Geijo-Barrientos	E	Emilio
Gelmi	Maria L	Maria Luisa
Gemignani	A	Angelo
George	A A Paul	Ajay Abisheck Paul
Georgiou	J	John
Gershwin	LA	Lisa-Ann
Ghelardini	C	Carla
Giannone	G	Gregory
Gibbs	A	Alan
Gilles	N	Nicolas
Gilly	WF	William F
Gilquin	B	Bernard
Giulianotti	Marc	Marc A
Glaaser	I	IW
Glick	SD	Stanley D

Goadsby	PJ	Peter J
Gobert	Geoffrey	Geoffrey N
Gomes	AV	Aldrin V
Gomez	MV	Marcus Vinicius
Gomez	Marcus V	Marcus Vinicius
Gomez	Renato S	Renato Santiago
Gondarenko	Elena	Elena A
Gonzalez	C	Carmen
Gonzalez	M	Monica
Gopel	S	SO
Gorini	C	Christopher
Goto	K	Kenichi
Gotti	C	Cecilia
Govarthanan	M	Muthusamy
Govarthanan	M.	Muthusamy
Gowd	K Hanumae	Konkallu Hanumae
Gowd	KH	Konkallu Hanumae
Gowd	Konkallu H	Konkallu Hanumae
Grady	SR	Sharon R
Graf	R	Roland
Grando	Sergei	Sergei A
Grassi	C	Claudio
Green	BR	Brad R
Green	AR	A Christopher
Greening	D	David
Gremski	Luiza H	Luiza Helena
Gribble	Fiona	Fiona M
Gribble	FM	Fiona M
Griffith	David	David A
Grinevich	Andrey	Andrey A
Grinevich	V	Vladimir P
Grinevich	VP	Vladimir P
Grinnell	AD	Alan D
Grohovaz	F	Fabio
Grossman	Y	Yoram
Grottesi	A	Alessandro
Gruber	Christian	Christian W
Gruber	HJ	Hermann J
Gruber	Hermann J.	Hermann J
Grundy	D	David
Grunnet	M	Morten
Guatimosim	C	Cristina
Guendisch	D	Daniela
Guerineau	Nathalie	Nathalie C
Guilloteau	D	Denis
Guimaraes	Andre L S	Andre Luiz Sena
Gulyas	J	Joszef
Gulyas	Josef	Jozsef
Gulyas	Joszef	Jozsef

Guo	Y	Ye
Guo	Zhan-yun	Zhan-Yun
Gutierrez	LM	Luis M
Gutierrez	Luis	Luis M
Gutierrez-Merino	C	Carlos
Haberberger	RV	Rainer Viktor
Habib	AM	Abdella Mohammed
Hackeng	Tilman	Tilman M
Haddad	Vidal	Vidal
Hagan	R	Rebecca H
Hagenacker	T	Tim
Hainaut	Pierre	Pierre L
Hainsworth	AH	Atticus H
Hainsworth	Atticus H.	Atticus H
Hamann	MT	Mark T
Hamann	Mark	Mark T
Hamann	Mark T.	Mark T
Hamilton	B	Brett
Hammond	Donna	Donna L
Han	HC	Hee Chul
Han	YuHong	Yu-Hong
Han	Yuhong	Yu-Hong
Handley	Thomas	Thomas N G
Hanson	GR	Glen R
Hansson	K	Karin
Hao	Marlene	Marlene M
Harrington	Andrea	Andrea M
Harris-Warrick	Ronald	Ronald M
Hartley	O	Oliver
Hascup	Erin R	Erin Rutherford
Hascup	Kevin	Kevin N
Hasegawa	T	Takafumi
Haubner	A	AJ
Hawrot	E	Edward
Haythornthwaite	A	Alison
He	Yawen	Ya-Wen
Heath	Christopher	Christopher J
Heimer	P	Pascal
Heinemann	SH	Stefan H
Heinemann	Stefan H.	Stefan H
Heintz	N	Nathaniel
Henao	F	Fernando
Henckaerts	Els	Els Jeanny V
Heralde	Frank M	Francisco M
Hernandez	M	Medardo
Hernandez-Guijo	JM	Jesus Miguel
Hernandez-Samano	Arisai C	Arisai C A
Herring	N	Neil
Herrington	J	James B

Herrington	James	James B
Hilber	K	Karlheinz
Hildebrand	ME	Michael E
Hilder	Tamsyn	Tamsyn A
Hill	JM	Justine M
Hillard	CJ	Cecilia J
Hillyard	DR	David
Himaya	SWA	Siddhihalu W A
Hinds	MG	Mark G.
Hinks	T	Timothy
Hirabayashi	T	Tetsuya
Hirota	K	Kazuyoshi
Hocking	Henry	Henry G
Hocking	HG	Henry G
Hofmann	F	Franz
Hofmann	Mackenzie	Mackenzie E
Hogg	R	Ron C
Hogg	RC	Ron C
Hoggard	Mickelene	Mickelene F
Hollmann	M	Michael
Hone	Arik	Arik J
Hone	AJ	Arik J
Hong	SK	Soo-Kyung
Hooper	D	David
Hoot	Michelle	Michelle R
Hopping	G	Gene G
Hopping	Gene	Gene G
Horinouchi	T	Takahiro
Horn	John P.	John P
Horvath	G	Gyongyi
Horvath	Martin	Martin P
Hossain	Mohammed	Mohammed Akhter
Hovius	R	Ruud
Hu	Honggang	Hong-Gang
Hu	Youtian	You-Tian
Huang	Feijuan	Fei-juan
Huang	Johnny	Johnny X
Huang	Shu Kuei	Shu-Kuei
Hubbard	Jeffrey	Jeffrey M
Huck	S	Sigismund
Hudecz	F	Ferenc
Hui	K	KY
Hur	EM	Eun Mi
Hutchinson	Dana	Dana S
Huxford	T	Tom
Huys	I	Isabelle
Ibanez-Tallon	I	Ines
Ichida	S	Seiji
Iengar	P	Prathima

Iijima	T	Toshihiko
Ikeda	M	Motoko
Ikeda	SR	Stephen R
Ikeda	Stephen	Stephen R
Illesinghe	Jayamini	Jayamini P
Imanishi	T	Takashi
Imhof	D	Diana
Imoto	K	Keiji
Imperial	J	Julita S
Imperial	JS	Julita S
Imperial	Julita	Julita S
Indurthi	Dinesh C	Dinesh C C
Inserra	Marco	Marco C
Irwin	RP	Robert P
Isa	T	Tadashi
Ishibashi	H	Hitoshi
Ishida	Y	Yukisato
Ishii	K	Kunio
Ishizuka	T	Toru
Ivanov	V	VT
Ivanov	IA	Igor A
Iwata	S	Seinosuke
Izzo	AA	Angelo A
Izzo	Angelo	Angelo A
Jabba	Sairam	Sairam V
Jackson	Kathryn	Kathryn A V
Jacobsen	R	Richard B
Jacobsen	RB	Richard B
Jacobsen	Richard	Richard B
Jahromi	Babak S.	Babak S
Jakubowski	JA	Jennifer A
Jamieson	Andrew	Andrew G
Jansen-Olesen	I	Inger
Jang	I-S	Il-Sung
Jang	IS	Il-Sung
Jara	Javier	Javier H
Jen	J	Jonas
Jenkins	Timothy P	Timothy P
Jennings	Ernest	Ernest A
Jensen	Anders	Anders Asbjorn
Jensen	Anders A	Anders Asbjorn
Jensen	Jonas	Jonas E
Jensen	LJ	Lars Jorn
Jeong	HJ	Hyo-Jin
Jeong	JY	Ji-Hyun
Jeong	SW	Seong-Woo
Jergova	S	Stanislava
Jerlhag	E	Elisabet
Jespersen	T	Thomas

Jiang	Z	Zherui
Jimenez	EC	Elsie C
Jimenez	Elsie	Elsie C
Jin	Ai-Hua (Jean)	Ai-Hua
Jin	AH	Ai-Hua
Jin	Ai-hua	Ai-Hua
Jin	Aihua	Ai-Hua
Jin	Zhichao	Zhi-Chao
Jobling	P	Phillip
Johnson	Philip	Philip L
Jois	SDS	Seetharama D S
Jonas	P	Peter
Jones	A	Alun
Jones	BE	Barbara E
Jones	SR	Sara R
Joshi	Dinesh	Dinesh C
Kaczorowski	GJ	Gregory J
Kalapothakis	E	Evanguedes
Kalia	Yogeshvar	Yogeshvar N
Kaneko	S	Shuji
Kang	TS	Tse Siang
Kannan	Rajesh R	Rajaretinam Rajesh
Kantor	Yuri	Yuri I
Kara	I	Irina
Karas	John	John A
Karas	JA	John A
Kasheverov	I	Igor E
Kasheverov	IE	Igor E
Kasheverov	I E	Igor E
Kasheverov	Igor	Igor E
Kashiwagi	A	Atsunori
Kashiwayanagi	M	Makoto
Kasten	Michael	Michael R
Kato	A	Akira
Katsura	M	Masashi
Kauferstein	S	Silke
Kaufmann	WA	Walter Anton
Kawada	T	Toru
Kawaguchi	A	Akinori
Kawakami	T	Takako
Kawano	Y	Yuhei
Kawasaki	H	Hideya
Kawasaki	HIdeya	Hideya
Keating	Damien	Damien J
Keays	D	DA
Kelber	O	Olaf
Kellar	KJ	Kenneth J
Kelley	WP	Wayne P
Kelly	Lauren	Lauren S

Kelstrup	Christian D	Christian Dahl
Kemenes	G	George
Kendel	Y	Yvonne
Kerekes	N	Nora
Kern	Steven	Steven E
Khammy	Makhala	Makhala M
Kho	KH	Kang Hee
Khoo	Keith	Keith K
Khruschov	Alexey	Alexey Yu
Khruschov	Alexey Y	Alexey Yu
Khwaja	M	Mirium
Kieffer	B	Bruno
Kilduff	TS	Thomas S
Kim	DH	Do H
Kim	KM	Kwang Myung
Kim	SJ	Sung Joon
Kim	Sung-Joon	Sung Joon
Kim	TJ	Tae Jin
Kim	Veronica	Veronica J
Kimura	S	Sadao
Kimura	T	Tomohiko
King	GF	Glenn F
King	Glenn	Glenn F
Kini	RM	R Manjunatha
Kirchner	Matthew	Matthew K
Kirk	Steven	Steven R
Kirkup	AJ	Anthony J
Klar	M	Maximilian
Klimis	H	Harry
Knapp	O	Oliver
Kobayashi	K	Kiyotaka
Koganei	H	Hajime
Ko	WH	Wun-Chang
Koh	Jennifer	Jennifer M S
Kohgo	Y	Yutaka
Kohlmeier	KA	Kristi A
Kohlmeier	Kristi A.	Kristi A
Kompella	Shiva	Shiva Nag
Kompella	Shiva N	Shiva Nag
Kong	ID	In Deok
Konno	K	Katsuhiro
Korf	Horst	Horst-Werner
Korkosh	Viacheslav	Viacheslav S
Korkosh	Vyacheslav	Viacheslav S
Korngreen	A	Alon
Koromyslova	Anna	Anna D
Koua	D	Dominique
Krais	Annette	Annette M
Krashia	Paraskevi	Paraskevi A

Krasteva-Christ	G	Gabriela
Kravchenko	Irina	Irina N
Kreis	ME	Martin E
Kreissl	S	Sabine
Krishnan	KS	Kozhalmannom S
Krishtal	O	Oleg A
Krishtal	OA	Oleg A
Kristensen	Jesper	Jesper Langgaard
Krizaj	I	Igor
Krnjevic	K	Kresimir
Kryukova	EV	Elena V
Kryukova	Elena	Elena V
Kuba	K	Kenji
Kubista	H	Helmut
Kubo	T	Tomoya
Kuch	U	Ulrich
Kudlacek	O	Oliver
Kudryashova	Ksenia	Ksenia S
Kudryavtsev	Denis	Denis S
Kudryavtsev	DS	Denis S
Kukolja	J	Juraj
Kukwa	W	Wojciech
Kumar	GS	Gopinatha Suresh
Kuo	J-S	Jon-Son
Kuo	Jinn Rung	Jinn-Rung
Kuo	Yuh-chi	Yuh-Chi
Kuok	WM	W-M
Kurniawan	Nyoman	Nyoman D
Kuryatov	A	Alexander
Kushmerick	C	Christopher
Lachapelle	P	Pierre
Lafourcade	Carlos	Carlos A
Lal	S	Sahil
Lalo	U	UV
Lamas	JA	J Antonio
Lamas	J. Antonio	J Antonio
Lambeau	G	Gerard
Lambert	DG	David G
Lambert	David	David G
Lambert	G	Gavin W
Lambert	Gavin	Gavin W
Lamthanh	H	Hung
Landa-Jaime	V	Victor
Lang	B	Bethan
Langeslag	M	Michiel
Lanuza	MA	Maria A
Lapied	B	Bruno
Lavergne	V	Vincent
Lavidis	NA	Nickolas A

Lawrence	N	Nicole
Layer	RT	Richard T
Le Caer	Jean-Piere	Jean-Pierre
Le Novere	N	Nicolas
Leao	RM	Ricardo M
Leao	Ricardo	Ricardo M
Lebaric	Z	Z N
Lebaric	ZN	Z N
Lebbe	Eline	Eline K M
Lecoq	A	Alain
Lee	C. Justin	C Justin
Lee	Reggie	Reggie Hui-Chao
Lee	SE	Sarah E
Lee	Sarah E.	Sarah E
Lee	SY	Sue Yeon
Lefebvre	RA	Romain A
Legros	C	Christian
Leipold	E	Enrico
Leite	R	Romulo
Lena	C	Clement
Leprince	J	Jerome
Leslie	FM	Frances M
Lester	HA	Henry A
Letchworth	SR	Sharon R
Levi	R	Roberto
Levoye	A	Angelique
Lewis	R	Richard James
Lewis	RJ	Richard James
Lewis	R J	Richard James
Lewis	Richard	Richard James
Lewis	Richard J	Richard James
Li	Fengmin	Feng-Min
Li	Wenxin	Wen-Xin
Li	Zhanchao	Zhan-Chao
Liang	SP	Songping
Liew	Yih-fong	Yih-Fong
Lihrmann	I	Isabelle
Lima	Pedro	Pedro A
Lindstrom	J	Jon M
Lindstrom	JM	Jon M
Lindstrom	Jon	Jon M
Lin	Tzu Yu	Tzu-Yu
Ling	C	CS
Lipkind	G	Gregory M
Lipscombe	D	Diane
Lipski	J	Janusz
Lirazan	M	MB
Liu	Yanli	Yan-Li
Liu	Yiqiao	Yi-qiao

Livett	B	Bruce G
Livett	BG	Bruce G
Livett	B G	Bruce G
Llames	Sara	Sara G
Lluisma	Arturo	Arturo O
Loane	David	David J
Lobanov	Alexander	Alexander V
Locatelli	V	Vittorio
Lockman	Paul	Paul R
Lok	YP	Y Phei
Lomax	Alan	Alan E
London	B	Barry
Long	L	Li-hong
Long	Paul	Paul F
Longhi	R	Renato
Lopez	MG	Manuela G
Lopez-Vera	E	Estuardo
Loughnan	M	Marion L
Loughnan	ML	Marion L
Loughnan	Marion	Marion L
Louiset	E	Estelle
Lovelace	ES	Erica S
Luchian	T	Tudor
Luetje	CW	Charles W
Lujan	R	Rafael
Lukacova	N	Nadezda
Lukas	RJ	Ronald J
Luke	M	Mark
Lukewich	Mark	Mark K
Lumb	Bridget	Bridget M
Luna-Ramirez	Karen	Karen S
Lund	TM	Trine Meldgaard
Lundeen	Rachel	Rachel A
Luo	Ji	Jing
Luo	Su-lan	Sulan
Luo	ZD	Z David
Lu	Cheng Wei	Cheng-Wei
Lusardi	T	Theresa
Lyeth	Bruce	Bruce G
Lyseng-Williamson	Katherine	Katherine A
Lyukmanova	Ekaterina	Ekaterina N
Ma	Xiaokuang	Xiao-Kuang
Ma	Xiao-kuang	Xiao-Kuang
MacDonald	Patrick	Patrick E
MacRaild	Christopher	Christopher A
Macdonald	RL	R Loch
Madeo	G	Graziella
Magistretti	J	Jacopo
Magyar	K	Kalman

Maillo	M	Maria
Maisonneuve	IM	Isabelle M
Malagoli	D	Davide
Malagon	M	Maria M
Maldonado	R	Rafael
Malhotra	A	Arun
Malomouzh	Artem	Artem I
Mameli	M	Manuel
Mandel	G	Gail
Mann	M	Matthias
Mans	Ben J.	Ben J
Mansbach	Rachael	Rachael A
Mansuelle	P	Pascal
Manzoni	OJ	Olivier J
Manzoni	Olivier	Olivier J
Marchetti	B	Bianca Maria
Mari	F	Frank
Marino	S	Silvia
Mark	Alan	Alan E
Marks	M	Michael J
Marks	MJ	Michael J
Marks	M J	Michael J
Marques	Antonio C	Antonio Carlos
Marrion	NV	Neil V
Martella	G	Giuseppina
Marthan	R	Roger
Martin	Jennifer L.	Jennifer L
Martin	M	Matthew P
Martin-Moutot	N	Nicole
Martin-Romero	FJ	Francisco Javier
Martinez	AC	Ana Cristina
Martinez	Ana	Ana Cristina
Martinez-Pinna	J	Juan
Martire	M	Maria
Martorana	A	Alessandro
Marubio	L	LM
Marvizon	JCG	Juan Carlos
Marx	UC	Ute C
Marx	Ute C.	Ute C
Mascia	MS	Maria Stefania
Masetto	S	Sergio
Maskos	U	Uwe
Massensini	AR	Andre Ricardo
Massensini	Andre	Andre Ricardo
Massensini	Andre R	Andre Ricardo
Massilia	GR	Gabriella Raybaudi
Matias	I	Ignacio R R
Matias	Ignacio R. R	Ignacio R R
Matsuda	T	Tomoki

Matsushima	K	Kayoko
Matteoli	M	Michela
Matthews	EA	Elizabeth A
Matthews	Elizabeth	Elizabeth A
Mattson	MP	Mark P
Matzno	S	Sumio
Maurice	T	Tangui
Mayer	AMS	Alejandro M S
Mayer	Alejandro	Alejandro M S
Maza	Julio	Julio R
Mazeh	Amna	Amna C
Mazzuoli	G	Gemma
McArthur	Jeff R	Jeffrey R
McArthur	Jeffrey	Jeffrey R
McArthur	JR	Jeffrey R
McCabe	RT	R Tyler
McCallum	SE	Sarah E
McClelland	D	David E
McClure-Begley	Tristan	Tristan D
McConnell	Matthew	Matthew J
McCool	BA	Brian A
McCool	Brian	Brian A
McCumber	D	Damon
McDonough	SI	Stefan I
McDougal	O	Owen M
McDougal	OM	Owen M
McDougal	Owen	Owen M
McIntosh	J M	J Michael
McIntosh	JM	J Michael
McIntosh	M	J Michael
McIntosh	Michael	J Michael
McKeith	I	Ian
McKeith	IG	Ian
McLachlan	EM	Elspeth M
McLeod	Malcolm D.	Malcolm D
McMahon	Kirsten	Kirsten L
McManus	Don	Donald P
McManus	OB	Owen B
McMaster	D	Denis
McWhinnie	Fergus	Fergus S
Meaney	D	David
Mebs	D	Dietrich
Meder	W	WP
Medina-Franco	Jose	Jose L
Meissner	Gabriel	Gabriel Otto
Melaun	C	Christian
Meldal	M	Morten
Melis	MR	Maria Rosaria
Melis	T	Tiziana

Melo	Eliane G	Eliane Goncalves
Melo	EG	Eliane Goncalves
Melo	Marilia	Marilia Martins
Melo	Marilia M	Marilia Martins
Mendelowitz	D	David
Mendoza	E	Ernesto
Mercado	F	Francisco
Mercuri	NB	Nicola Biagio
Mercuri	Nicola B	Nicola Biagio
Meredith	Andrea	Andrea L
Mergler	S	Stefan
Meriney	SD	Stephen D
Merola	F	Fabienne
Messing	RO	Robert O
Messing	Robert	Robert O
Meunier	FA	Frederic A
Meyer	Christopher	Christopher P
Meyer	Erin	Erin L
Mezo	G	Gabor
Michel	K	Klaus
Michel	PP	Patrick Pierre
Middleton	Leah	Leah J
Mikoshiba	K	Katsuhiko
Miles	LA	Luke Anthony
Miles	Luke	Luke Anthony
Miljanich	G	George P
Millard	EL	Emma L
Milne	TJ	Trudy J
Miloslavina	Alesia	Alesia A
Minakata	H	Hiroyuki
Minami	K	Kazuhisa
Miranda	L	Les P
Mishra	Santosh	Santosh K
Miwa	JM	Julie M
Miwa	Julie	Julie M
Miyamoto	N	Norimasa
Moczydlowski	EG	Edward G
Mogg	A	AJ
Moglie	Marcelo	Marcelo J
Moglie	Marcelo J.	Marcelo J
Mohammadi	Sarasa	Sarasa A
Mohammed	Afrah	Afrah Eltayeb
Mohammed	Afrah E.	Afrah Eltayeb
Mohammed	Afrah E	Afrah Eltayeb
Mok	K. Hun	K Hun
Mok	KH	K Hun
Mokler	DJ	David J
Mokler	David J.	David J
Moldavan	MG	Mykhaylo G

Molenaar	P	Peter
Molgo	J	Jordi J
Molgo	Jordi	Jordi J
Moller	C	Carolina
Momiyama	T	Toshihiko
Montano	LM	Luis M
Montero	M	Mayte
Montiel	C	Carmen
Moore	J	Jessi
Mora	D	Daniel
Morales	MA	Miguel Angel
Morales	Miguel A	Miguel Angel
Morales	Rodrigo	Rodrigo A V
Moran	O	Oscar
Morandini	Andre C	Andre Carrara
Mordvintsev	DY	Dmitry Y
Moretta	M	Melissa
Moretti	M	Milena
Morimoto	S	Shin-ichi
Morita	H	Hiromitsu
Mori	H	Hidezo
Morlighem	Jean-Etienne	Jean-Etienne R L
Mortensen	M	Martin
Moser	A	A James
Moser	T	Tobias
Motin	L	Leonid
Mozar	Christine	Christine A
Mozar	CA	Christine A
Mueller	A	Alexander
Mugnaini	M	Manolo
Muizelaar	JP	J Paul
Mulder	RJ	Roger J
Muldoon	PP	Pretal P
Mule	F	Flavia
Mullan	M	Michael
Mulvenna	Jason	Jason P
Munhall	A	Adam
Murakami	M	Manabu
Murakami	N	Noboru
Muraki	Y	Yo
Murayama	T	Takanori
Musachio	J	JL
Musci	Giovanni	Giovannni
Mutafova-Yambolieva	VN	Violeta N
Nada	SA	Somaia A
Nagashima	M	Michio
Nagasu	T	Takeshi
Naidoo	V	Vinogran

Nair	Sudarslal Sadasivan	Sudarslat Sadasivan
Nakagawasai	O	Osamu
Nakajima	M	Mayumi
Nakamura	M	Mitsuhiro
Nakao	Y	Yoichi
Nam	Hannah	Hannah H
Napier	IA	Ian A
Naranjo	D	David
Nashmi	R	Raad
Nathani	Neelam	Neelam M
Natochin	Yu V	Yury V
Navarra	P	Pierluigi
Needham	K	Karina
Neelands	TR	Torben R
Neher	E	Erwin
Neves	Jorge	Jorge L B
Nevin	S	Simon T
Nevin	ST	Simon T
Nevin	S T	Simon T
Nevin	Simon	Simon T
Newton	PM	Philip M
Newton	Philip	Philip M
Ngo	Shyuan	Shyuan T
Nguyen	Tam	Tam H
Nichols	Colin	Colin G
Nichols	RA	Robert A
Nicholson	Graham	Graham M
Nicke	A	Annette
Nielsen	CK	Carsten K
Nielsen	J	JS
Nielsen	KJ	Katherine J
Nielsen	Lau	Lau D
Nieto	Juan J.	Juan J
Nimmervoll	B	Benedikt
Noakes	Peter	Peter G
Noebels	JL	Jeffrey L
Noebels	Jeffrey	Jeffrey L
Nonaka	K	Kiku
Nordberg	A	Agneta
Norenberg	W	Wolfgang
North	RA	R Alan
Norton	RS	Raymond S
Norton	Raymond	Raymond S
Nunes	Kenia	Kenia P
Nurnberg	B	Bernd
O'Donnell	Tracy	Tracey
O'Leary	Kathryn	Kathryn T
O'Mara	Megan	Megan L
O'Neill	C	Christopher

O'Neill	Heidi	Heidi C
Oba	Y	Yuichi
Obermair	G	G J
Obermair	GJ	G J
Obis	T	Teresa
Oda	M	Masataka
Oh	SB	Seog Bae
Ohkuma	S	Seitaro
Ohmori	H	Harunori
Ohtake	A	Atsuko
Ojomoko	Lucy	Lucy O
Okada	M	Motohiro
Okada	S	Shoshiro
Okada	Yohei	Yasunobu
Olesen	J	Jes
Olesen	SP	Soren P
Oliva	C	Carolina
Oliveira	Joaquim M.	Joaquim Miguel
Oliveira	Joaquim M	Joaquim Miguel
Oliveira	L	Laura JF
Oliveira	Sara M	Sara Marchesan
Olivera	BM	Baldomero M
Olivera	Baldomera M	Baldomero M
Olivera	Baldomero	Baldomero M
Olsen	Jesper	Jesper V
Omatsu-Kanbe	M	Mariko
Onaka	T	Tatsushi
Ono	F	Fumihito
Ono	K	Kentaro
Oppenheim	RW	Ronald W
Orensanz	LM	Luis M
Orr-Urtreger	A	Avi
Ortiz	E	Ernesto
Osipov	Alexey	Alexey V
Osipov	Aleksei	Alexey V
Osipov	AV	Alexey V
Ostroumov	V	Vitaly
Ottaviani	E	Enzo
Otuki	Michel	Michel Fleith
Pacher	P	Pal
Paci	M	Maurizio
Page	CP	Clive Peter
Pakhomov	Andrei	Andrei G
Palant	E	Elka
Pan	Q	Quan
Pan	Yi	Yaping
Panatier	A	Aude
Pankratov	Y	YV
Pannaccione	A	Anna

Paolini-Bertrand	M	Marianne
Parameswaran	N	Neeraja
Pardos-Blas	Jose R	Jose Ramon
Parente	V	Valeria
Parsadaniantz	S Melik	Stephane Melik
Patel	Dharmeshkumar	Dharmeshkumar Jethalal
Patel	R	Ryan
Patil	Nitin	Nitin A
Patzlaff	NE	Natalie E
Paulino	N	Niraldo
Peckys	D	Diana B
Peckys	Diana	Diana B
Peckys	Diana B.	Diana B
Peers	C	Chris
Peigneur	S	Steve
Pemberton	K	KE
Pennington	MW	Michael W
Perez	Edwin	Edwin G
Perez	XA	Xiomara A
Perrotti	L	Linda
Perry	DC	David C
Perry	E	EK
Perry	R	RH
Perugini	Matthew	Matthew A
Petrou	S	Steven
Phillips	PEM	Paul E M
Phillips	Paul	Paul E M
Phillips	Paul E. M.	Paul E M
Picciotto	MR	Marina R
Picciotto	Marina R.	Marina R
Pickering	Anthony	Anthony E
Picolo	G	Gisele
Piggott	M	MA
Pilowsky	PM	Paul Martin
Pilowsky	Paul M	Paul Martin
Pimenta	Adriano M. C.	Adriano M C
Pimenta	AMC	Adriano M C
Pimenta	DC	Daniel C
Pinborg	Lars H	Lars Hageman
Pineda	JC	Juan Carlos
Pinto	FM	Francisco M
Pinto	Francisco M.	Francisco M
Piriz	J	Joaquin
Pisani	A	Antonio
Pivavarchyk	M	Marharyta
Platt	Randall	Randall J
Plazas	PV	Paola V
Plazas	Paola	Paola V
Polidano	M	MA

Polticelli	F	Fabio
Poncer	JC	Jean Christophe
Portales-Casamar	E	Elodie
Portaro	F	Fernanda
Portaro	FCV	Fernanda
Possani	LD	Lourival D
Post	Michael	Michael R
Poulter	CD	C Dale
Poulter	Russell	Russell T M
Pouzat	C	Christophe
Pozo	Maria	Maria J
Prashanth	JR	Jutty Rajan
Prado	MAM	Marco Antonio Maximo
Prado	Marco A M	Marco Antonio Maximo
Prado	Marco Antonio M	Marco Antonio Maximo
Prado	Vania F	Vania Ferreira
Prator	Cecilia	Cecilia A
Prevette	D	David
Price-Carter	M	Marian
Priego	M	Mercedes
Prieto	D	Dolores
Priller	J	Josef
Prorok	M	Mary
Pszczolkowski	MA	Maciej A
Puillandre	N	Nicolas
Pupo	AS	Andre Sampaio
Purcell	Anthony	Anthony W
Purcell	AW	Anthony W
Qadri	F	Firdausi
Qin	N	Ning
Quik	M	Maryka
Raghothama	S	Srinivasarao
Ragnarsson	L	Lotten
Raimondi	L	Laura
Raiteri	M	Maurizio
Rajesh	Rajaian P	Rajaian Pushpabai
Rajnavolgyi	E	Eva
Ramaswami	M	Mani
Ramiro	Iris Bea	Iris Bea L
Ranasinghe	Shiwanthi	Shiwanthi L
Rajesh	RP	Rajaian Pushpabai
Ramirez	JM	Jan-Marino
Ray	M	MA
Recio	P	Paz
Recio-Pinto	E	Esperanza
Reeh	Peter W.	Peter W
Reeves	M	Matthew
Reid	Robert	Robert C
Reimann	F	Frank

Reis	Rui L.	Rui L
Renart	J	Jaime
Reynolds	Eric	Eric C
Rhim	H	Hyewhon
Rich	MM	Mark M
Richardson	M	Michael
Richter	K	Katrin
Rigo	Flavia K	Flavia Karine
Rigual	R	Ricardo
Rittenhouse	AR	Ann R
Rivera	A	Alicia
Rivera	L	Luis
Rivera-Reyes	R	Reginaldo
Rivier	J	Jean E
Rivier	JE	Jean E
Rivier	JEF	Jean E
Rivier	Jean	Jean E
Robbe	D	David
Robinson	Samuel	Samuel D
Rodriguez-Farre	E	Eduard
Rodriguez-Farre	E.	Eduard
Rodriguez-Tapia	Eileen	Eileen S
Roeper	J	Jochen
Rohlf	F. James	F James
Rola	R	Rafal
Rolando	B	Barbara
Roman-Gonzalez	Sergio A	Sergio Agustin
Roman-Gonzalez	Sergio A.	Sergio Agustin
Romano Silva	Marco A	Marco Aurelio
Romano-Silva	MA	Marco Aurelio
Romano-Silva	Marco A	Marco Aurelio
Romeo	C	Cristina
Roper	Stephen	Stephen D
Rorsman	P	Patrik
Rosa	Juliana M	Juliana Martins
Rosado	Isabel R	Isabel Rodrigues
Rosenbaum	T	Tamara
Rosengren	K. Johan	K Johan
Rosengren	KJ	K Johan
Rossato	Mateus F	Mateus Fortes
Rossi	F	Francesco Mattia
Rossi	FM	Francesco Mattia
Rossi	ML	Maria Lisa
Rostene	W	William
Roubos	EW	Eric W
Roussel	C	Christian
Rozhkova	A	AM
Rudd	John A.	John A
Rugiero	F	Francois

Rutledge	Malcolm	Malcolm T
Rutten	K	Kris
Rybin	Matt	Matthew J
Rychkov	Grigori	Grigori Y
Rykov	Vladimir	Vladimir A
Sabareesh	V	Varatharajan
Safronova	Valentina	Valentina G
Saito	H	Hiroko
Saito	M	Maki
Sakuma	Y	Yasuo
Salas	R	Ramiro
Saleem	Moin	Moin A
Salehi	A	Albert
Salgado	H	Humberto
Salminen	O	Outi
Salvador-Reyes	Lilibeth	Lilibeth A
Salzet	M	Michel
Samigullin	Dmitry	Dmitry V
Samigullin	Dmitry V.	Dmitry V
Sanchez-Prieto	J	Jose
Sand	O	Olav
Sandall	D	David W
Sandall	DW	David W
Sandall	David	David W
Sanjakdar	SS	Sarah S
Sanders	KM	Kenton M
Sandkuhler	J	Jurgen
Sandtner	W	Walter
Sanfeliu	C	Coral
Sanjakdar	Sarah	Sarah S
Sansom	MSP	Mark S P
Santafe	MM	Manel M
Santafe	Manel	Manel M
Santiago	Ana R	Ana Raquel S
Santicioli	P	Paolo
Santos	AD	Ameurfina D
Sarma	SP	Siddhartha P
Sarma	Siddhartha	Siddhartha P
Sasakawa	T	Tomoki
Sasaki	S	Shigekazu
Saska	Z	Zsuzsanna
Satoh	E	Eiki
Sato	K	Kazuki
Sauer	Susanne	Susanne Katharina
Scadden	Mickl	Mick'l
Scamps	F	Frederique
Scanlon	Martin	Martin J
Schafer	Laurel	Laurel L
Scheenen	W	Wim

Schemann	M	Michael
Schemm	R	Rudolf
Schiavo	G	Giampietro
Schilaty	Nate	Nathan
Schilaty	Nathan	Nathan D
Schinina	M Eugenia	Maria Eugenia
Schinina	ME	Maria Eugenia
Schioth	Helgi	Helgi B
Schirinzi	T	Tommaso
Schmachtenberg	O	Oliver
Schmidt	JJ	James J
Scholze	A	Alexandra
Scholze	P	Petra
Schousboe	A	Arne
Schroder	W	Wolfgang
Schroeder	CI	Christina I
Schroeder	Christina	Christina I
Schroeder	Christina I.	Christina I
Schulz	J	Joseph R
Schulz	JR	Joseph R
Schwarting	R	Rainer
Schwarting	RAINER	Rainer
Schwartz	A	AD
Schwarz	Stephan	Stephan K W
Sciamanna	G	Giuseppe
Scornik	FS	Fabiana S
Seagar	M	Michael
Seagar	MJ	Michael
Secondo	A	Agnese
Selvankumar	T	Thangaswamy
Selvatici	R	Rita
Sendtner	M	Michael
Serebryakova	Marina	Marina V
Serov	Dmitriy	Dmitriy A
Servent	D	Denis
Sethupathy	S	Subramaniam
Seyedi	N	Nahid
Shafer	Steven	Steven L
Shafer	TJ	Timothy J
Shafer	Timothy	Timothy J
Shah	M	Mala
Shaitan	KV	Konstantin V
Shao	Xiaoxia	Xiao-Xia
Shao	XX	Xiao-Xia
Sharp	T	Trevor
Sharpe	IA	Iain A
Shelukhina	Irina	Irina V
Shelukhina	IV	Irina V
Shen	J	Jintao

Sheng	Z	Zhenyu
Shepherd	Nicholas	Nicholas E
Shi	L	Liying
Shibuya	I	Izumi
Shigemoto	R	Ryuichi
Shinozuka	K	Kazumasa
Shishido	T	Toshiaki
Shoudai	K	Kiyomitsu
Shulepko	Mikhail	Mikhail A
Siciliano	Cody	Cody A
Sihra	TS	Talvinder S
Sikdar	SK	Sujit K
Silva	AP	Ana Paula
Silva	Carla	Carla Maria O
Silva	Carla M O	Carla Maria O
Silva	Cassia R	Cassia Regina
Silva	Juliana F	Juliana Figueiredo
Silva	Juneo F	Juneo Freitas
Silver	RB	Randi B
Simone	Luiz Ricardo L.	Luiz Ricardo L
Singh	Brij	Brij B
Siniavin	Andrei	Andrei E
Siniscalchi	A	Anna
Sirockin	F	Finton
Sivilotti	Lucia	Lucia G
Sixma	TK	Titia K
Skalicky	Jack	Jack J
Smit	AB	August B
Smith	Alexander	Alexander C W
Smith	BJ	Brian J
Smith	D	DH
Smith	Jennifer J	Jennifer Jean
Smith	MT	Maree T
Smith	McHardy	McHardy M
Smith	PA	Paul A
Smith	Paul	Paul A
Smout	Michael	Michael J
Smulski	Cristian	Cristian R
Smyth	LM	Lisa M
Smyth	N	Neil
Smythe	Mark	Mark L
Snutch	TP	Terrance P
Sobey	CG	Christopher G
Sochivko	D	Dmitry
Soderpalm	B	Bo
Son	Lina	Lina V
Souza	Alessandra H	Alessandra Hubner
Sparatore	F	Fabio
Spat	A	Andras

Spirova	EN	Ekaterina N
Sporning	A	Annett
Staheli	S	ST
Staras	K	Kevin
Steen	H	Hanno
Stenflo	J	Johan
Stephens	Gary	Gary J
Stevens	K	Kate
Stevenson	Kate	Kate E
Stewart	James	James C
Stiles	JR	Joel R
Stockdill	Jennifer	Jennifer L
Stocker	M	Martin
Stoecklin	R	Reto
Storer	RJ	Robin James
Storey	KB	Kenneth B
Stout	Kristen	Kristen A
Striessnig	J	Joerg
Strosznajder	Joanna	Joanna B
Su	Ruibin	Rui-Bin
Succu	S	Salvatora
Sudweeks	Sterling	Sterling N
Sugimachi	M	Masaru
Sulzenbacher	G	Gerlind
Sumithran	SP	Sangeetha P
Summers	RJ	Roger J
Sun	Xiaoping	Xiao-Ping
Sun	Zhihua	Zhi-hua
Surges	R	Rainer
Surowy	CS	Carol S
Susilawathi	Ni M	NI Made
Suwansa-ard	S	Saowaros Mai
Suzuki	T	Tsutonu
Svensson	L	Lennart
Swartz	KJ	Kenton J
Swartz	Timothy	Timothy D
Swedberg	Joakim	Joakim E
Sweedler	JV	Jonathan V
Swensen	Andrew	Andrew M
Symington	SB	Steven B
Szabo	G	Gabor
Szucs	G	Geza
Szulczyk	P	Pawel
Tabakmakher	Valentin M.	Valentin M
Tabakmakher	VM	Valentin M
Tabor	Alethea	Alethea B
Taccola	G	Giuliano
Tack	J	Jan
Tadano	T	Takeshi

Taglialatela	M	Maurizio
Taguchi	K	Kyoji
Takahara	A	Akira
Takahashi	E	Eiki
Takahashi	M	Masataka
Takahashi	T	Tomoyuki
Takeuchi	K	Koh
Takigawa	M	Morikuni
Talley	Todd	Todd T
Tamiya	E	Eiichi
Tan	Roger	Roger S
Tan-No	K	Koichi
Tanaka	H	Hiromasa
Tanaka	K	Keiko
Tanaka	N	Naoko
Tarasova	Olga S.	Olga S
Tassone	A	Annalisa
Taupignon	A	Anne
Taylor	K	KL
Taylor	P	Palmer
Taylor-Clark	Thomas	Thomas E
Tayo	Lemmuel	Lemmuel L
Tecuapetla	F	Fatuel
Teichert	RW	Russell W
Teichert	Russell	Russell W
Terpinskaya	TI	Tatiana I
Tekinay	AB	Ayse Begum
Terlau	H	Heinrich
Terpinskaya	Tatiana	Tatiana I
Thakur	Suman	Suman S
Theilmann	DA	David A
Thomas	L	Linda
Thompson	Philip	Philip E
Thompson	SM	Scott M
Thompson	Stuart	Stuart A
Tian	Changlin	Chang-Lin
Tieleman	DP	D Peter
Tieleman	D.Peter	D Peter
Tietze	Alesia	Alesia A
Tikhonov	DB	Denis B
Tikhonov	Denis	Denis B
Timmermann	DB	Daniel B
Timperley	Christopher	Christopher M
Todt	H	Hannes
Toledo-Aral	JJ	Juan J
Tomas	J	Josep
Tomas	M	Marta
Torok	TL	Tamas L
Torres	A	Angela

Torres	J	Josep Lluis
Torres	M	Magdalena
Torres	Natalia	Natalia S
Tostes	Rita	Rita C
Toth	I	Istvan
Tournier	JM	Jean-Marie
Townsend	A	Amanda L
Tran	Hue	Hue Ngoc Thi
Tran	Hue N T	Hue Ngoc Thi
Tranberg	C Elisabet	Charlotte Elisabet
Trevisan	Gabriela	Gabriela GT
Tringham	Elizabeth	Elizabeth W
Troncone	LRP	Lanfranco R P
Troncone	Lanfranco R. P.	Lanfranco R P
Tsetlin	V	Victor I
Tsetlin	VI	Victor I
Tsetlin	V I	Victor I
Tsetlin	Victor	Victor I
Tsien	RW	Richard W
Tsigelny	I	Igor
Tsujimura	A	Atsushi
Tsuneki	H	Hiroshi
Tuck	Kellie	Kellie L
Tuominen	R	RK
Turner	Matt	Matthew W
Twede	Vernon	Vernon D
Tyndall	JDA	Joel D A
Tyndall	Joel	Joel D A
Tynngard	N	Nahreen
Tytgat	J	Jan
Tzartos	Socrates	Socrates J
Tzeng	Woan-fang	Woan-Fang
Uchida	T	Takahiro
Uchitel	OD	Osvaldo D
Uchitel	Osvaldo	Osvaldo D
Ueberheide	Beatrix	Beatrix M
Ueda	H	Hiroshi
Ueta	Y	Yoichi
Uhrenholt	TR	Torben R
Uhrenholt	Torben R.	Torben R
Ulens	C	Chris
Upert	G	Gregory
Uramura	K	Kazuhide
Uray	K	Katalin
Urbano	FJ	Francisco J
Uribe	Juan E.	Juan E
Usowicz	Maria	Maria M
Utkin	Y	Yuri N
Utkin	YN	Yuri N

Utkin	Yu N	Yuri N
Utkin	Yuri	Yuri N
Utkin	Yuri N.	Yuri N
Valli	P	Paolo
Van Renterghem	C	Catherine
Vanden Berghe	P	Pieter
Vanthuyne	N	Nicolas
Vargas	MH	Mario H
Vargas	Mario H.	Mario H
Vaughan	Christopher	Christopher W
Vaughan	CW	Christopher W
Veenstra	Jan	Jan Adrianus
Veenstra	Jan A	Jan Adrianus
Vega	R	Rosario
Veith	Paul	Paul D
Venkatesha	MA	M Achanna
Venter	DJ	Deon J
Verderio	C	Claudia
Vernier	P. Thomas	P Thomas
Vetter	Douglas E.	Douglas E
Vieira	LB	Luciene Bruno
Vieira	Luciene B	Luciene Bruno
Viero	C	Cedric
Vigh	J	Jozsef
Vijayaragavan	K	Kausalia
Vijayasarathy	M	Marimuthu
Villegas	E	Elba
Viniegra	S	Salvador
Viola	Maria	Maria Francesca
Vizi	ES	E Sylvester
Voge	H	Heiko
Volsen	S	SG
Vulfius	CA	Catherine A
Vulfius	Catherine	Catherine A
Vyskocil	F	Frantisek
Wada	T	Tetsuyuki
Wade	JD	John D
Wade	John	John D
Wageman	CR	Charles R
Wagstaff	JD	John D
Wagstaff	John	John D
Wakamori	M	Minoru
Walker	Andrew	Andrew A
Walker	C	Craig S
Walker	CS	Craig S
Wallace	MJ	Melisa J
Wallace	Melisa	Melisa J
Walsh	J	JP
Wang	C-I Anderson	Ching-I Anderson

Wang	Ching-I A	Ching-I Anderson
Wang	CZ	Cheng Z
Wang	Conan	Conan K L
Wang	Conan K	Conan K L
Wang	G	Gang
Wang	Huili	Hui-Li
Wang	Su-Jane	Su Jane
Wang	Su-jane	Su Jane
Wang	Yujia	Yu-Jia
Wanke	E	Enzo
Watkins	M	Maren
Watson	M	Maureen
Webb	RC	Robert Clinton
Weinreich	D	Dan
Wen	H	Hua
Wennemuth	G	Gunther
West	P	PJ
Westermann	Jan-C	Jan-Christoph
Westfall	TC	Thomas C
White	Fletcher	Fletcher A
White	H. Steve	H Steve
White	HS	H Steve
Whiteaker	P	Paul
Whitt	Joshua	Joshua P
Wickenden	AD	Alan D
Wiedenmann	B	Bertram
Wilbanks	Sigurd	Sigurd M
Wilcox	KS	Karen S
Wills	Zachary	Zachary P
Williamson	NA	Nicholas A
Wilson	David	David T
Winkel	KD	Kenneth D
Winkel	Kenneth D	Kenneth D
Winkel	Kenneth D.	Kenneth D
Winkfein	R	RJ
Witzemann	V	Veit
Wolf	JA	John A
Wolf	M	Miriam
Wolfart	J	Jakob
Wolfe	BB	Barry B
Wong	Clarence T T	Clarence Tsun Ting
Wonnacott	S	Susan
Wood	JN	John N
Woodward	Taylor	Taylor J
Woycechowsky	Kenneth	Kenneth J
Wray	S	Susan
Wright	CE	Christine E
Wright	Christine	Christine E
Wright	E	Elizabeth M

Wu	Fangming	Fang-Ming
Wu	Xuechen	Xue-Chen
Wu	Yingliang	Ying-Liang
Wunder	C	Cora
Xie	JY	Jennifer Y
Xie	Jennifer	Jennifer Y
Xu	B	Bing
Xu	F	Fang
Xu	AL	Anlong
Xue	L	Liang
Xue	T	Tian
Yada	T	Toshihiko
Yadav	J	Jhillu Singh
Yaksh	TL	Tony L
Yaksh	Tony	Tony L
Yamanaka	A	Akihiro
Yamazaki	K	Koji
Yamazaki	T	Toji
Yamboliev	IA	Ilia A
Yan	C	Cui
Yanai	K	Kazuhiko
Yanchanka	Tatsiana	Tatsiana L
Yanchanka	TL	Tatsiana L
Yang	Weishan	Wei-Shan
Yasuda	RP	Robert P
Yasuda	T	Takahiro
Yawo	H	Hiromu
Ye	JH	Jiang-Hong
Yearwood	T	Thomas
Yokotani	K	Kunihiko
Yon	L	Laurent
Yoon	SH	Shin Hee
Yorgason	Jordan	Jordan T
Yorgason	JT	Jordan T
Yoshida	M	Masashi
Yoshida	S	Shigeru
Yoshikami	D	Doju
Yoshinaga	T	Takashi
Young	Heather	Heather M
Young	Neil	Neil D
Yu	C	Chunlu
Yu	Eizadora	Eizadora T
Yuan	Duoduo	Duo-Duo
Zajicek	J	Jaroslav
Zaki	Nazar	Nazar M
Zambelli	Vanessa	Vanessa O
Zamora-Bustillos	R	Roberto
Zamponi	GW	Gerald W
Zamponi	Gerald	Gerald W

Zanardi	A	Alessio
Zaveri	Nur T	Nurulain T
Zaveri	NT	Nurulain T
Zeilhofer	HU	Hanns Ulrich
Zelepuga	Elena	Elena A
Zelepuga	EA	Elena A
Zeng	J	Jie
Zhan	JB	Jin-Biao
Zhan	Jin-biao	Jin-Biao
Zhan	Jinbiao	Jin-Biao
Zhang	Min-Mm	Min-Min
Zhang	Minmin	Min-Min
Zhang	Tongliang	Tong-Liang
Zhangsun	Dongting	Dong-ting
Zhangsun	Donting	Dong-ting
Zhao	Ruiming	Rui-Ming
Zhao-Shea	Rubing	Rubin
Zheng	Yiwu	Yufei
Zhmak	M	Maxim N
Zhmak	MN	Maxim N
Zhmak	M N	Maxim N
Zhmak	Maxim	Maxim N
Zhorov	BS	Boris S
Zhorov	Boris	Boris S
Zhorov	Boris S.	Boris S
Zhou	HM	H Mimi
Zhou	Xiaowei	Xiao-Wei
Zhu	J	Jiang
Ziganshin	Rustam	Rustam H
Zoli	M	Michele
Zorn	S	Stefan
Zou	X-Y	Xiao-Yong
Zucca	G	Giampiero
Zugasti-Cruz	A	Alejandro
Zweckstetter	M	Markus
da Silva	Juliana	Juliana Figueira
da Silva	JF	Juliana Figueira
de Araujo	Aline D	Aline Dantas
de Curtis	M	Marco M
de Curtis	Marco	Marco M
de Diego	Antonio M G	Antonio Miguel G
de Dios	AC	Angel C
de Haro	L	Luc
de Kater	AnneliesW	Annelies
de Veer	Simon	Simon J
de la Cotera	EPH	Edgar P Heimer
de Biasi	M	Mariella
de Leon-Nava	Marco A.	Marco A
de Lima	ME	Maria Elena

de Marco	L	Luiz
de Marinis	Yang	Yang Zhang
de Nucci	G	Gilberto
de Pascual	R	Ricardo
de Pra	Samira	Samira Dal-Toe
de Waard	M	Michel
van Elk	R	Rene
van Luijtelaar	G	Gilles
van der Donk	Wilfred	Wilfred A
von Kugelgen	I	Ivar
de Souza	AH	Alessandra Hubner
